# Racial disparities in breast cancer treatment patterns and treatment related adverse events

**DOI:** 10.1038/s41598-023-27578-4

**Published:** 2023-01-22

**Authors:** Nickolas Stabellini, Jennifer Cullen, Lifen Cao, John Shanahan, Nelson Hamerschlak, Kristin Waite, Jill S. Barnholtz-Sloan, Alberto J. Montero

**Affiliations:** 1 Graduate Education Office, Case Western Reserve University School of Medicine, Cleveland, OH USA; 2 Department of Hematology-Oncology, University Hospitals/Seidman Cancer Center, Breen Pavilion - 11100 Euclid Ave, Cleveland, OH 44106 USA; 3 Faculdade Israelita de Ciências da Saúde Albert Einstein, Hospital Israelita Albert Einstein, São Paulo, SP Brazil; 4 Department of Population and Quantitative Health Sciences, Case Western Reserve University School of Medicine, Cleveland, OH USA; 5 Case Western Reserve University/Case Comprehensive Cancer Center, Cleveland, OH USA; 6 Cancer Informatics, University Hospitals/Seidman Cancer Center, Cleveland, OH USA; 7 Oncohematology Department, Hospital Israelita Albert Einstein, São Paulo, SP Brazil; 8 Trans-Divisional Research Program (TDRP), Division of Cancer Epidemiology and Genetics (DCEG), National Cancer Institute, National Institutes of Health, Bethesda, MD USA; 9 Center for Biomedical Informatics and Information Technology (CBIIT), National Cancer Institute, National Institutes of Health, Bethesda, MD USA

**Keywords:** Breast cancer, Oncology, Public health

## Abstract

The main objective of this work was to perform a comprehensive analysis and provide a race-stratified epidemiological report accounting for differences in treatment patterns and treatment related adverse events in Non-Hispanic women with breast cancer (BC). The cohort included women ≥ 18 years diagnosed with *in-situ*, early-stage, and late-stage BC (2005–2022). Treatment patterns included: surgery, breast radiation, chemotherapy, endocrine therapy, or biologic therapy. Treatment related adverse events were: chemotherapy complications, cardiovascular toxicities, immune-related adverse events, psychological affectations, or cognitive decline/dementia. The influence of race on the outcomes was measured via Cox proportional-hazards models. We included 17,454 patients (82% non-Hispanic Whites [NHW]). Most of the patients had a Charlson Comorbidity Score between 1 and 2 (68%), and TNM stage I (44.5%). Surgery was performed in 51.5% of the cases, while 30.6% received radiotherapy, 26.4% received chemotherapy, 3.1% received immunotherapy, and 41.2% received endocrine therapy. Non-Hispanic Blacks (NHB) had a lower probability of undergoing breast cancer surgery (aHR = 0.92, 95% CI 0.87–0.97) and of being prescribed endocrine therapy (aHR = 0.83, 95% CI 0.79–0.89), but a higher probability of receiving adjuvant radiotherapy (aHR = 1.40, 95% CI 1.29–1.52). Moreover, NHBs had lower risk of being diagnosed with psychological issues (aHR = 0.71, 95% CI 0.63–0.80) but a higher risk for cognitive decline/dementia (aHR = 1.30, 95% CI 1.08–1.56). In conclusion, NHB women diagnosed with BC were less likely than NHW to undergo curative intent surgery or receive endocrine therapy, and had a higher risk of cognitive decline/dementia after cancer treatment. Public policy measures are urgently needed which equalize access to quality healthcare for all patients and that promote a learning healthcare system which can improve cancer outcomes.

## Introduction

Breast cancer (BC) is the most frequently diagnosed malignancy among women globally, and the leading cause of cancer death, and in 2020 was responsible for 2,261,419 new cases (11.7% of the total), and 684,996 deaths (6.9% of the total)^1^. In the United States (US) alone, there will be an estimated 287,850 new cases (15% of the total) and 43,250 deaths (7.1% of the total) in 2022^2,3^. According to the National Cancer Institute (NCI), approximately 13% of women will be diagnosed with BC at some point during their life^3^.

With a 5-year relative survival of approximately 91%, treatment of BC involves one or a combination of surgery (lumpectomy or mastectomy), radiotherapy, and/or neoadjuvant or adjuvant systemic therapy (endocrine therapy, chemotherapy, and/or anti-HER2 directed antibody therapy or immunotherapy), depending on staging, pathological, and molecular features^4,5^. Despite cure rates and efficacy, BC treatments are accompanied by acute and late effects^6–9^. Common effects include both physical symptoms such as fatigue, erythema, pneumonia, heart problems, neutropenic infection, nausea, vomiting, diarrhea, neuropathy, alopecia and menopausal symptoms, as well as psychological symptoms, mainly involving anxiety and depression^7–9^. Late toxicities of breast cancer treatments can also include permanent cardiovascular and bone marrow toxicities. The combination of adverse effects, both acute and late, is responsible for changes in quality of life—an important prognostic factor^6–8^.

In the published literature, the reality of racial cancer disparities is well documented^10–12^. In BC, Black patients, when compared to other groups, are more likely to be diagnosed at more advanced stages, have limited access to quality health care, and have a higher risk of having triple negative breast cancer (TNBC)—the BC subtype with the poorest prognosis^10,13–16^. Consequently, because of these myriad factors Black women with breast cancer have a higher mortality risk. It is believed that part of the BC disparities in survival outcomes are explained by disparities in treatment as Black patients are less likely to receive adequate treatment and more likely to experience significant treatment delays compared to White patients^17,18^. Reports also indicate that, for cancer as overall, racial minorities are at increased risk for poorer health outcomes, e.g. hospitalization, health-care associated infections, and emergency department (ED) visits^19–21^.

Despite racial disparities in BC outcomes and in access to treatment being well documented in the medical literature, to our knowledge there is scant information on whether racial disparities also exist with respect to treatment patterns and treatment related adverse events in BC patients. We hypothesize that there are differences in the incidence of adverse treatment effects which might contribute to racial disparities in BC outcomes. The primary objective of this study is to perform a comprehensive analysis and provide an epidemiological report stratified by race accounting for treatment patterns and treatment adverse events in Non-Hispanic women with BC.

## Methods

The study setting was the University Hospitals (UH) Seidman Cancer Center (Northeast Ohio, US). All patient data were obtained from the UH data repository based on the CAISIS platform, which consists of an open-source, web-based cancer data management system composed by disparate sources of cancer patient data (i.e., Soarian, NGS Labs, Sunrise Clinical Manager, Tumor Registry, Via Oncology, OnCore, MosiaQ, PRO tools, and others)^22–24^. All patient records were de-identified, and all analyses were performed in accordance with relevant guidelines and regulations, respecting the Declaration of Helsinki. The study with the waiver of the informed consent was approved by the University Hospitals of Cleveland Institutional Review Board (IRB). All the information obtained from the UH database was subsequently complemented with electronic health record (EHR) information captured via EMERSE (Electronic Medical Record Search Engine) in order to obtain the most accurate and complete information per patient, avoiding high missingness^25^.

**Figure 1:**
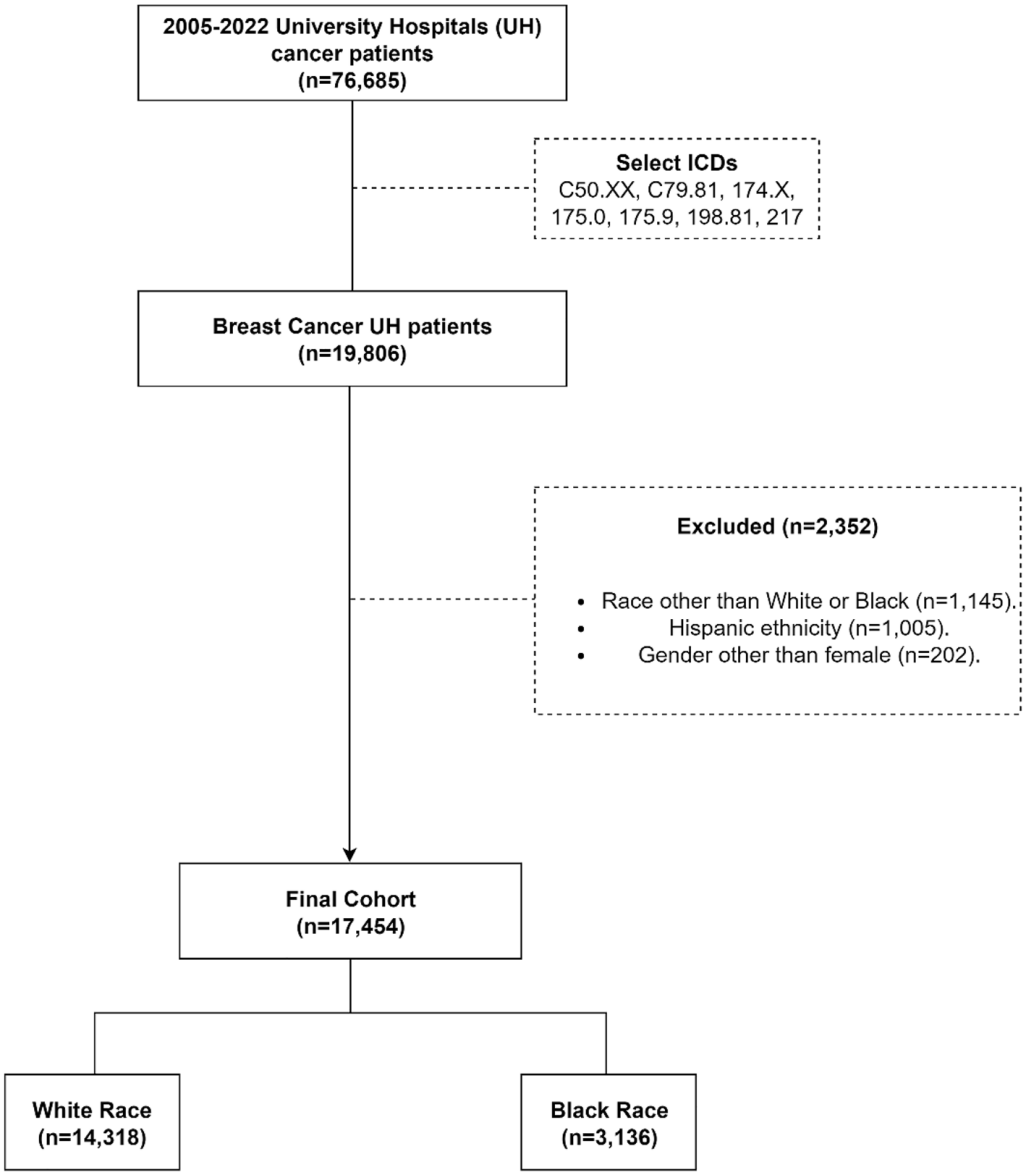
Study consort diagram detailing inclusion and exclusion criteria for Breast Cancer University Hospitals (UH) population (2005–2022). The final cohort included 17,454 patients, of which 3136 (18%) self-reported as Black.

The initial cohort included women ≥ 18 years diagnosed with *in-situ*, early-stage, or late-stage breast cancer (henceforth, breast cancer; determined using Tumor Registry (TR) or Electronic Medical Record (EMR) International Classification of Diseases [ICD] 9/10 codes: C50.XX, C79.81, 174.X, 175.0, 175.9, 198.81, 217, with X standing for any integer) between 01/01/2005 and 03/31/2022^26^. Patients were excluded from the analysis if they had race other than Black or White, ethnicity different than Non-Hispanic (due to the low number of Hispanic patients in the dataset), and gender different than female. Hispanics were excluded due very low patient numbers, while other races were excluded to focus the analysis exclusively on disparities on treatment related toxicities in White vs. Black BC patients. The cohort selection consort diagram is presented in Fig. 1.

### Outcomes

Study outcomes included: (I) treatment and time-to-treatment following index breast cancer diagnosis; (II) the diagnosis and time-to-event of a treatment adverse event.

### Covariates

Demographics, BC pathological characteristics, treatment patterns, and treatment adverse events data were obtained for all eligible patients. The demographic characteristics included: age at breast cancer diagnosis, self-reported race (White, Black), smoking status (yes, no, former, unknown), and Charlson comorbidity index^27^. Tumor characteristics included: date of BC diagnosis, hormone receptor status (estrogen receptor [ER], progesterone receptor [PR]) and HER2 status (positive or negative as per ASCO/CAP guidelines), histological type (ductal carcinoma, ductal or lobular carcinoma in-situ [DCIS/LCIS], and other), and TNM staging group (stage 0–IV)^28–30^.

Treatment patterns examined in this study included: treatment adherence (number of appointments per patient and % of appointments attended), surgery (mastectomy or lumpectomy), breast radiation (right, left, or bilateral), chemotherapy, endocrine therapy, or immunotherapy (HER-2 or PDL1 antibody therapy). The use of single or multiple treatment modalities were accounted. The medical treatments included as covariates included: anthracyclines, PIK3CA/mTOR inhibitors, HER2-targeted agents, ER antagonists, LHRH agonists, aromatase inhibitors (AI), or other novel biologic therapies. Time-to-treatment variables were extracted based on the date of BC diagnosis and the date of the first treatment (surgery, radiation, or systemic therapy). The medications included in each category are described in Supplemental Table I.

Treatment adverse events were extracted on the basis of ICD 9/10 codes, where only diagnoses occurring after the date of first treatment were considered. Complications from any treatment included: cognitive decline or dementia (yes, no), or the presence of psychological a disorder (yes, no) and characterization of the psychological disorder (depression, anxiety, or bipolar disorder). Chemotherapy complications included those most frequently reported in the literature^22,23,31^. Complications from immunotherapy were described as immune-related adverse events (irAEs) (yes, no) and the specific irAEs^22,23,31^. The ICD codes and categorizations are summarized in Supplemental Table 1.

### Statistical analysis

The population was described via percentages, median and interquartile range (IQR). All descriptive and inferential statistics were race-stratified to examine racial differences. The Pearson Chi-Square test was used to compare categorical variables. Data distribution assumptions for continuous variables were confirmed using histograms and the Kolmogorov–Smirnov test, followed by Student’s T-tests for normally distributed factors and non-parametric Kruskal–Wallis tests for non-normal distributed factors. The influence of race on type of treatment received, and treatment adverse events was assessed via Hazard Ratios (HR) or adjusted Hazard Rations (aHR) with 95% confidence intervals (CIs), using univariable and multivariable Cox proportional-hazards models, after confirming the model’s assumptions. Patients were censored according to the last follow-up date and the models for treatment adverse events were performed only in patients who received the respective treatment. Sensitivity analysis was performed in patients diagnosed after 2015 to mitigate the effect of temporal changes.

The multivariable selected were those that achieved a *p* < 0.10 in univariable analyses for the primary outcome and those deemed to have clinical importance by study investigators. Independent variable correlations were checked by correlation plots, and those variables found to be correlated were not included simultaneously in the final multivariable models. A *p*-value < 0.05 was considered significant in the final models, and missing values were not included in the final analysis. All analyses were performed using RStudio software^32^. We used the STROBE cohort checklist when writing our report^33^.

### Ethical approval

Patient records were deidentified, and the study was approved by the University Hospitals of Cleveland Institutional Review Board (IRB).

## Results

### Population

Using data from 2005 to 2022, we analyzed 17,454 BC Non-Hispanic women with a BC diagnosis. The cohort’s median age was 63 (interquartile range [IQR] 53–73) years, with a predominance of Non-Hispanic Whites (NHWs) (82%). Most of the patients had a Charlson Comorbidity Score between 1 and 2 (68%), and TNM stage I (44.5%). Surgery was performed in 51.5% of patients, while 30.6% received radiotherapy, 26.4% received chemotherapy, 3.1% received immunotherapy, and 41.2% received endocrine therapy.

### Racial disparities in demographics and tumor characteristics

Among 17,454 patients analyzed, 18% were Non-Hispanic Blacks (NHBs). NHB were followed-up for a median of 4.4 years, while NHW were followed-up for a median of 8.1 years. Compared to NHWs, NHB patients had a significantly lower median age at diagnosis (62, IQR 52–72 vs. 63, IQR 53–73, *p* = 0.001), and a significantly higher: probability of a prior smoking history (13.5% vs. 9%, *p* < 0.001), proportion of women with Charlson comorbidity scores ≥ 5 (21.4% vs. 9.2%, *p* < 0.001), Ductal carcinoma (48.8% vs. 39.9%, *p* < 0.001), and stage IV (6.4% v 4.9%, *p* < 0.001, Table 1).

**Table 1:** Breast Cancer University Hospitals (UH) population (2005–2022) description and comparison stratified by race in demographics and tumor characteristics.

	Breast cancer UH population (n = 17,454)		
	Black	White	*p* value
	3136 (18%)	14,318 (82%)	
Age at diagnosis—median (IQR)	62 (52–72)	63 (53–73)	0.001
Year of diagnosis—n (%)			
2005–2010	669 (21.3%)	2890 (20.2%)	< 0.001
2010–2015	914 (29.1%)	3622 (25.3%)	
> 2015	1553 (49.5%)	7806 (54.5%)	
Smoking status—n (%)			
Smoker	283 (13.5%)	902 (9%)	< 0.001
Never smoker	1205 (57.7%)	6232 (62.5%)	
Former smoker	601 (28.5%)	1840 (28.5%)	
Unknown	1047	4,344	
Charlson score—n (%)			
1–2	1599 (51%)	10,278 (71.8%)	< 0.001
3–4	865 (27.6%)	2716 (19%)	
≥ 5	672 (21.4%)	1324 (9.2%)	
Histology—n (%)			
DCIS/LCIS	164 (5.2%)	532 (3.7%)	< 0.001
Ductal	1529 (48.8%)	5706 (39.9%)	
Other	1443 (46%)	8080 (56.4%)	
Stage—n (%)			
0	348 (15.3%)	1174 (12.8%)	< 0.001
I	843 (37.1%)	4250 (46.4%)	
II	655 (28.8%)	2425 (26.5%)	
III	282 (12.4%)	864 (9.4%)	
IV	145 (6.4%)	451 (4.9%)	
Unknown	863	5154	
ER + − n (%)	1364 (43.5%)	5998 (41.9%)	0.1
PR + − n (%)	1201 (38.3%)	5358 (37.4%)	0.36
HER2 + − n (%)	119 (3.8%)	454 (3.2%)	0.08
Triple positive—n (%)	52 (1.7%)	229 (1.6%)	0.87

A total of 17,454 patients were analyzed, with 3,136 (18%) self-reporting as Black. *IQR* interquartile range.

### Racial disparities in treatment patterns

When comparing treatment rates in NHBs vs. NHWs (Table 2), we found that women from the first group had higher rates of surgery (58% vs. 50.1%, *p* < 0.001), radiotherapy (42.1% vs. 28%, *p* < 0.001), chemotherapy (34.6% vs. 24.6%, *p* < 0.001), hormone therapy (42.6% vs. 40.9%), immunotherapy (4.1% vs. 2.8%, *p* < 0.001), and combined therapy (including combined modality and combined systemic therapy). Stratifying the analysis by specific medications (Table 2), NHBs were prescribed both anthracycline containing (11.4% vs. 7.4%, *p* < 0.001), and non-anthracycline containing chemotherapy regimens (22.1% vs. 15.5%, *p* < 0.001) at a significantly higher rates. We also found that NHB were prescribed endocrine therapies at higher rates: aromatase inhibitors (12% vs. 7.7%, *p* < 0.001), LHRH agonists (2.6% vs. 1.9%, *p* = 0.01), ER antagonists (6.2% vs. 4.4%, *p* < 0.001), as well as other biologic therapies (6.7% vs. 5.3%, *p* < 0.001).

**Table 2:** Breast Cancer University Hospitals (UH) population (2005–2022) description and comparison stratified by race in treatment patterns. IQR = interquartile range.

	Breast cancer UH population (n = 17,454)		
	Black	White	*p* value
	3136 (18%)	14,318 (82%)	
Surgery—n (%)	1819 (58%)	7167 (50.1%)	< 0.001
Mastectomy—n (%)	490 (15.6%)	1790 (12.5%)	< 0.001
Lumpectomy—n (%)	583 (18.6%)	1771 (12.4%)	< 0.001
Time to surgery (days)—median (IQR)	42 (27–106)	34 (21–62)	< 0.001
Radiotherapy (R)—n (%)	1320 (42.1%)	4015 (28%)	< 0.001
Right side only radiotherapy—n (%)	371 (11.8%)	1136 (7.9%)	< 0.001
Left side only radiotherapy—n (%)	315 (10%)	967 (6.8%)	< 0.001
Time to radiotherapy (days)—median (IQR)	204 (99–287)	138 (77–253)	< 0.001
Chemotherapy (C)—n (%)	1085 (34.6%)	3528 (24.6%)	< 0.001
Time to chemotherapy (days)—median (IQR)	70 (37–112)	62 (36–95)	< 0.001
Hormone therapy (H)—n (%)	1335 (42.6%)	5853 (40.9%)	0.08
Time to hormone therapy (days)—median (IQR)	138 (72–245)	126 (72–218)	0.003
Immunotherapy (I)—n (%)	129 (4.1%)	406 (2.8%)	< 0.001
Time to immunotherapy (days)—median (IQR)	68 (43–127)	59 (34–128)	0.44
Combined therapy			
C + R − n (%)	718 (22.9%)	1,558 (10.9%)	< 0.001
I + R − n (%)	87 (2.8%)	206 (1.4%)	< 0.001
H + R − n (%)	733 (23.4%)	2,285 (16%)	< 0.001
H + C + R − n (%)	383 (12.2%)	1,008 (7%)	< 0.001
H + C + R + I − n (%)	39 (1.2%)	111 (0.8%)	0.01
Agents			
Anthracyclines—n (%)	357 (11.4%)	1,053 (7.4%)	< 0.001
Non-anthracycline cytotoxic chemotherapy—n (%)	694 (22.1%)	2,214 (15.5%)	< 0.001
PIK3CA/mTOR inhibitors—n (%)	4 (0.1%)	9 (0.1%)	0.4
Aromatase inhibitors—n (%)	375 (12%)	1,098 (7.7%)	< 0.001
LHRH agonists—n (%)	82 (2.6%)	270 (1.9%)	0.01
ER antagonists—n (%)	196 (6.2%)	631 (4.4%)	< 0.001
Newer therapies—n (%)	211 (6.7%)	752 (5.3%)	< 0.001
Appointments per patient—median (IQR)	10 (5–23)	8 (4–17)	< 0.001
% of appointments attended—median (IQR)	66 (44–80)	69 (50–85)	< 0.001

By contrast, NHBs had longer delays for surgery (median of 42 days [IQR 27–106] vs. 34 days [IQR 21–62]), radiotherapy (median of 204 days [IQR 99–287] vs. 138 days [77–253]), chemotherapy (median of 70 days [IQR 37–112] vs. 62 days [IQR 39–95]), as well as time to initiation of endocrine therapy (median of 138 days [IQR 72–245] vs. 126 days [IQR 72–218], Table 2). Despite a higher number of appointments per patient (10 [IQR 5–23] vs. 8 [IQR 4–17]), NHBs had lower median appointment completion rates (66% appointments attended [IQR 44–80] vs. 69% [IQR 50–85]).

### Racial disparities in treatment related adverse events

Analyzing treatment related adverse events (Table 3), considering only patients that received the respective treatments, NHBs had higher rates of chemotherapy related complications (20.9% vs. 12.2%, *p* < 0.001), including: cardiomyopathy, diarrhea/enteritis, fatigue, nausea/vomiting, neuropathy, lung disease, pain, dehydration/hypovolemia, rash, and infusion reactions, as well as higher reported rates of cognitive decline/dementia (13.6% vs. 6.7%, *p* < 0.001). Although no differences were seen in the incidence of overall immune related toxicities (irAEs), NHBs had higher rates cardiac toxicties acute myocardial infarction (3.1% vs. 0.2%, *p* = 0.01), and pneumonitis (7.8% vs. 2%, *p* = 0.003).

**Table 3:** Breast Cancer University Hospitals (UH) population (2005–2022) description and comparison stratified by race in treatment adverse events, including chemotherapy complications, irAEs (immune-related adverse events), psychological affections, and cognitive decline/dementia. *IQR* interquartile range; *MI* myocardial infarction; *AKI* acute kidney injury.

	Breast cancer UH population (n = 17,454)		
	Black	White	*p* value
**Chemotherapy complications—n (%)**	**227 (20.9%)**	**431 (12.2%)**	** < 0.001**
Adverse reaction—n (%)	11 (1%)	21 (0.6%)	0.21
Cardiomyopathy—n (%)	14 (1.3%)	16 (0.5%)	0.005
Diarrhea/enteritis—n (%)	41 (3.8%)	64 (1.8%)	< 0.001
Fatigue—n (%)	54 (5%)	80 (2.3%)	< 0.001
Nausea/vomiting—n (%)	62 (5.7%)	94 (2.7%)	< 0.001
Steatohepatitis—n (%)	6 (0.6%)	19 (0.5%)	1
Neuropathy—n (%)	27 (2.5%)	37 (1%)	< 0.001
Thrombocytopenia—n (%)	2 (0.2%)	6 (0.2%)	1
Lung disease—n (%)	35 (3.2%)	27 (0.8%)	< 0.001
Pain—n (%)	53 (4.9%)	54 (1.5%)	< 0.001
Anemia—n (%)	10 (0.9%)	15 (0.4%)	0.08
Agranulocytosis—n (%)	46 (4.2%)	156 (4.4%)	0.86
Mouth sore—n (%)	0	2 (0.1%)	1
Dehydration/hypovolemia—n (%)	38 (3.5%)	57 (1.6%)	< 0.001
Renal failure—n (%)	1 (0.1%)	0	0.53
Rash—n (%)	7 (0.6%)	1	< 0.001
Infusion reaction—n (%)	6 (0.6%)	5 (0.1%)	0.03
**IRAES**—**n (%)**	**47 (36.4%)**	**153 (37.7%)**	**0.06**
Anemia—n (%)	10 (7.8%)	36 (8.9%)	0.83
Thrombocytopenia—n (%)	2 (1.6%)	20 (4.9%)	0.15
Leukopenia—n (%)	9 (7%)	45 (11.1%)	0.23
Hypothyroidism—n (%)	11 (8.5%)	43 (10.6%)	0.60
Hyperthyroidism—n (%)	2 (1.6%)	4 (1%)	0.95
Hypophysitis/PGA—n (%)	3 (2.3%)	5 (1.2%)	0.63
Hyper/hypo- parathyroidism—n (%)	0	4 (1%)	0.58
AKI—n (%)	8 (6.2%)	14 (3.4%)	0.26
Neuritis—n (%)	4 (3.1%)	11 (2.7%)	1
Hepatitis—n (%)	0	3 (0.7%)	0.76
Colitis—n (%)	3 (2.3%)	16 (3.9%)	0.55
Pancreatitis—n (%)	3 (2.3%)	1 (0.2%)	0.07
Mucositis—n (%)	1 (0.8%)	3 (0.7%)	1
Arrhythmia—n (%)	7 (5.4%)	28 (6.9%)	0.70
Acute MI—n (%)	4 (3.1%)	1 (0.2%)	0.01
Myocarditis—n (%)	7 (5.4%)	24 (5.9%)	1
Pericarditis—n (%)	0	2 (0.5%)	1
Cardiomyopathy—n (%)	3 (2.3%)	9 (2.2%)	1
Pneumonitis—n (%)	10 (7.8%)	8 (2%)	0.003
Type I diabetes—n (%)	2 (1.6%)	3 (0.7%)	0.75
Meningitis—n (%)	0	0	-
Encephalitis, myelitis, encephalomyelitis—n (%)	0	0	-
Vitiligo—n (%)	1 (0.8%)	0	0.54
**Psychological affections**—**n (%)**	**598 (26.4%)**	**2,505 (27.5%)**	**0.30**
Depression—n (%)	381 (16.8%)	1,516 (16.6%)	0.86
Anxiety—n (%)	440 (19.4%)	1,893 (20.8%)	0.15
Bipolar Disorder—n (%)	36 (1.6%)	96 (1.1%)	0.04
**Cognitive decline/dementia**—**n (%)**	**308 (13.6%)**	**608 (6.7%)**	** < 0.001**

Bold values indicates the psychological affections category is composed of depression + anxiety + bipolar disorder.

### Association between race and treatment or treatment adverse events

**Figure 2:**
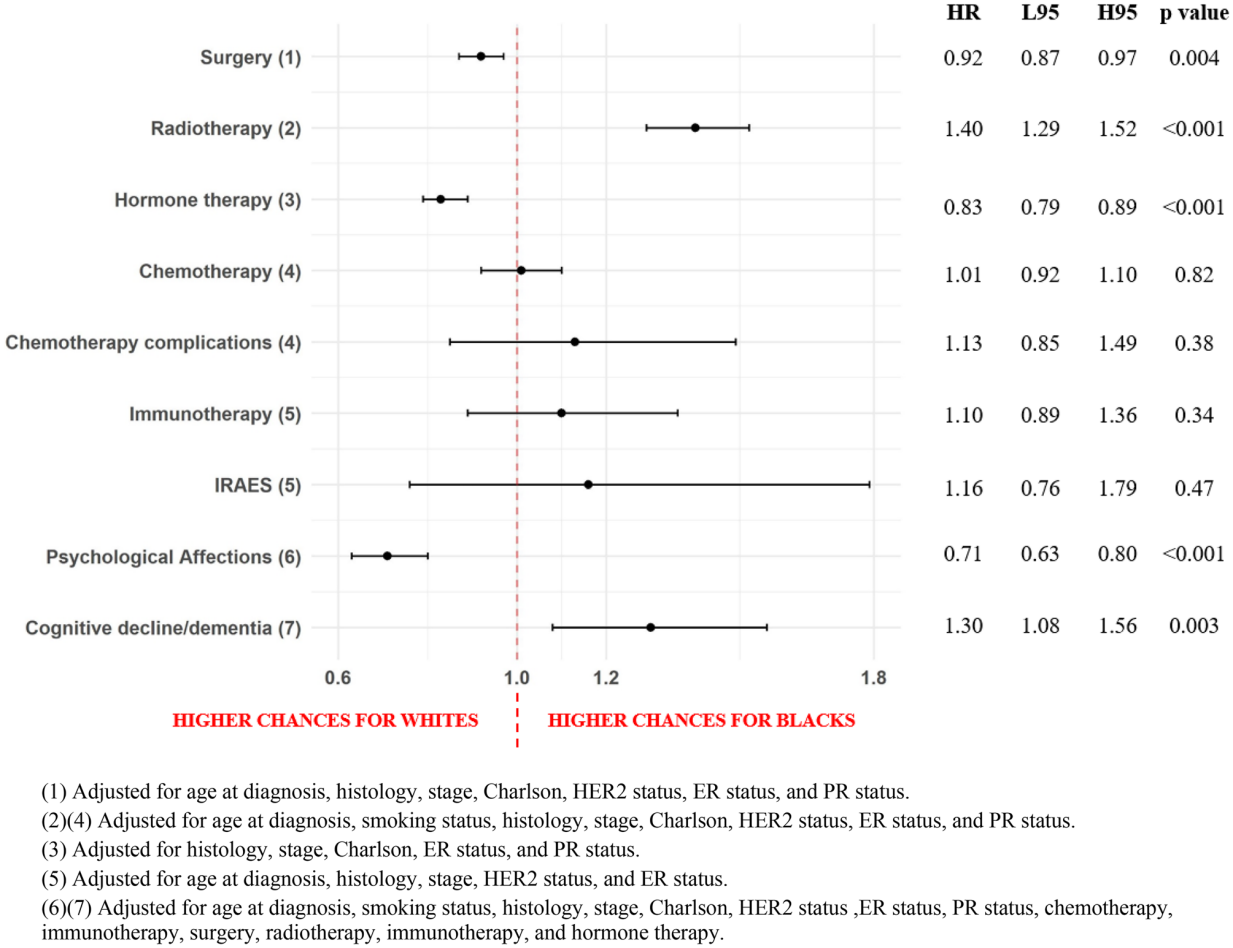
Forest plot detailing association between race and treatment patterns or treatment adverse events for Breast Cancer University Hospitals (UH) population (2005–2022). Results are presented in hazard ratios (HR) for Blacks, lower 95% confidence intervals (L95), higher 95% confidence intervals (H95), and *p*-value.

Multivariable cox proportional-hazards regressions (Fig. 2) revealed that, using NHWs as reference, NHBs had a lower probability of undergoing curative intent breast cancer surgery (aHR = 0.92, 95% CI 0.87–0.97) and of being prescribed endocrine therapy (aHR = 0.83, 95% CI 0.79–0.89), but a higher probability of receiving adjuvant radiotherapy (aHR = 1.40, 95% CI 1.29–1.52). For treatment related adverse mental health events, NHBs had lower risk of being diagnosed with psychological issues (aHR = 0.71, 95% CI 0.63–0.80) but a higher risk for cognitive decline/dementia (aHR = 1.30, 95% CI 1.08–1.56).

Sensitivity analysis considering patients diagnosed after 2015 (n = 8,635) showed similar results (Supplemental Table II).

## Discussion

The primary objective of this study was to perform a comprehensive analysis and provide an epidemiological exploration of racial disparities in treatment patterns and treatment related adverse events in Non-Hispanic women diagnosed with BC. With a retrospective design, our analyses included over 17,000 patients over a 17-year period and found that, when compared to NHW, NHB had lower probability of undergoing curative intent breast cancer surgery or of being prescribed endocrine therapy. At the same time NHB had lower rates of adherence to outpatient medical visits. Furthermore, we found that NHB compared to NHW had a 19% lower risk of being diagnosed with a psychological disorder, but a 30% higher risk of being diagnosed with cognitive decline/dementia after BC treatment. Our findings are important because it adds another dimension to BC racial disparities described in the literature, and point to the need for more personalized care and the development of public policies that equalize access to quality healthcare for minorities to mitigate poor outcomes.

In addition to our main findings, we also found that, besides breast cancer diagnosis occurring at younger age, NHBs had worse baseline health (characterized by higher levels of Charlson comorbidity score, and higher rates of smokers), and were more often diagnosed at advanced stages. Moreover, NHBs had higher rates of all treatment types alone or combined and a higher number of appointments per patient.

Racism is a central topic when analyzing racial disparities, and can manifest in a variety of ways which can impact health directly or indirectly^34^. Several published studies have demonstrated a direct correlation between self-reported personally-mediated racism and negative physical and mental health outcomes^35,36^. For example, inequities in income, education, employment and living standards, can greatly impact individual living environments and exposure to risk and protective factors^35,37–40^. Health consequences also occurs due to physical violence and stress pathways, which have negative psychological and physiological impacts^35^. In addition, racism is present in healthcare both in institutions and providers, leading to difficulties both in accessing and obtaining quality care^35,41^.

Historically, BC incidence is lower in Black when compared to White patients, however temporal trends show an increasingly incidence in the first group, and a stable pattern in the second group, probably as a result of improvement in health care access by Black patients^42^. Black women are diagnosed with BC at younger ages, and later stages, in addition to higher risks of lymph node or distant metastasis^43,44^. Several factors may explain these differences, which are consistent with our findings. There are biologic factors at play: Black women are diagnosed with TNBC at higher rates, are more likely to have somatic TP53 mutation, and less likely to have somatic PIK3CA mutations^45–49^. There are also environmental factors, such as social determinants of health (SDOH) which greatly influence cancer outcomes. Unemployment rates are higher in the Black community and are correlated with less job-based medical insurance and lower financial stability, creating barriers to health care access and leading to a delayed screening and diagnosis^45,50,51^.

For treatment, as stated above, Black women have higher rates of unemployment and a higher financial insecurity, which can negatively impact timely access to health care. This adds to the neighborhood context, as poor neighborhoods tends to be distant from health services^45^.

Prior studies have also previously reported that Black women experience greater time to BC treatment delays , and are more likely to experience very long treatment delays, and, even when they have access to treatment are less likely to receive treatments that are in accordance with national evidence based guidelines^52–54^. Furthermore, prior studies have also shown that Black BC patients are more prone to receive lower chemotherapy doses and greater likelihood of treatment modifications^55–58^. Our treatment patterns findings are consistent with the racial cancer disparities literature. The lower appointment completion rates in NHB may be linked to the barriers to accessing healthcare due to adverse SDOH and racism. We found higher rates of treatment in the NHB population, likely due to the more advanced breast cancer stage and histological type of disease at the time of initial diagnosis, factors that may also be the explanation for lower chances of surgery and hormone therapy, and higher chances of radiotherapy reported. Interestingly, previous studies have shown that NHB in the general population are less likely to be offered or receive radiotherapy^59^.

Prior cancer disparities work has not examined to what extent differences in the frequency or types of adverse effects of systemic treatments among NHB and NHW BC patients contributes to disparities in BC outcomes. Some studies have previously reported racial disparities in adverse drug events (ADE) in the general population being treated with anticoagulants, diabetic medications, and opioids^60^. We found that NHBs are at higher risk of cognitive decline/dementia, and cardiovascular (CV) subtypes of treatment adverse events. Higher risk of cognitive decline/dementia for NHB in the general population have been reported, but there are no published reports on the differential impact of breast cancer treatment on cognitive decline among NHB patients^61^. These poorer BC outcomes for NHB women and differences in treatment patterns suggest rather stark differences in the quality of cancer care which is a function of race^19,62–66^.

The racial disparities existent in demographics, treatment patterns, and treatment adverse events ultimately leads to disparities in BC mortality. Due to evolutions in treatment and screening, BC mortality reduced a 40% overall in the US since 1990^67^. However, differences between races in BC mortality still existing, with an estimated 40% higher risk in NHBs than in NHW^45,67,68^.

This study has several limitations. Our institutional database is EMR-based, and some of the information in the EMR may be incomplete. As a study in a single institution, some patients may have been to lost follow-up or sought emergency care at other institutions, and the lack of available data could have impacted our analysis. Adverse event rates were based on EMR ICD codes and can be underreported. Some of the ICD codes utilized to identify treatment related adverse effects are not treatment-specific and therefore may not be treatment-related. The extended timeframe employed can encompass generational changes in treatment, which we mitigated with sensitivity analysis of patients diagnosed after 2015. Also, we used a database that integrates disparate sources and includes detailed and longitudinal information on each patient, rarely seen in other databases. Finally, as an oncology center, we maintain a close follow-up with patients who usually come to our emergency (ED).

In summary, we found not only racial disparities in curative intent BC surgery, as well as important differences in endocrine and/or chemotherapy, but we also identified differences between NHB and NHW BC patients in the frequency and types of treatment related adverse events they experience. Taken together, racial disparities in BC outcomes is related not only to socioeconomic differences and differences in access to care, but also differences in the types of curative interventions they undergo and differences in treatment related toxicities.

## Supplementary Information

Supplementary Information.

## Data Availability

University Hospitals (UH) Seidman Cancer Center database is available at University Hospitals Cleveland Medical Center and has access restricted to researchers with IRB approval. Data are however available from the corresponding author upon reasonable request.
