# Supplementary material for: Racial disparities in breast cancer treatment patterns and treatment related adverse events
Source: Sci Rep. 2023 Jan 22;13:1233. doi: 10.1038/s41598-023-27578-4 (PMC9868122; doi:10.1038/s41598-023-27578-4)
Supplement: Supplementary file 1 — Supplementary Information. [file 41598_2023_27578_MOESM1_ESM.docx]

**Racial disparities in female breast cancer treatment patterns and treatment adverse events**

**Supplemental Material**

**Supplemental Table I.** Class of medications and respective medications included in each category. ICD 9/10 codes included for the subtypes of chemotherapy complications, irAEs, psychological affections, and cognitive decline/dementia.

| **Class of Medications** | **Medications Included** |
| --- | --- |
| HER2-Agents | Hyaluronidase, Trastuzumab, Lapatinib, Neratinib, Fam-Trastuzumab Deruxtecan, Ado-Trastuzumab Emtansine, Margetuximab, Pertuzumab, Hyaluronidase, Tucatinib, Dacomitinib |
| Aromatase Inhibitors | Letrozole, Anastrozole, Exemestane, Testolactone. |
| LHRH Agonists | Leuprolide, Goserelin Triptorelin. |
| Anthracyclines | Epirubicin, Daunorubicin, Doxorubicin, Idarubicin. |
| Non-anthracycline cytotoxic chemotherapy | Cyclophosphamide, Doxorubicin, Paclitaxel, Docotaxel, Fluorouracil, Capecitabine, Carboplatin, Methotrexate, Epirubicin, Cisplatin, Vinorelbine, Gemcitabine, Ixabepilone, Eribulin, Mitaxantrone, Mitomycin. |
| ER Antagonists | Tamoxifen, Raloxifene, Toremifene, Fulvestrant |
| PIK3CA/mTOR inhibitors | Alpelisib, Everolimus |
| Newer therapies | Trastuzumab, Lapatinib, Naratinib, Margetuximab, Pertuzumab, Tucatinib, Dacomitinib, Olaparib, Abemaciclib, Palbociclib, Ribociclib, Alpelisib, Pembrolizumab, Atezolizumab |
| **Chemotherapy** | |
| **Complication** | **ICD Codes** |
| Chemotherapy adverse reaction | T45.1, T88.7XXA; E933.1; 995.20; E947.9 |
| Chemotherapy-induced cardiomyopathy | I42.X; 425.4 |
| Chemotherapy-induced diarrhea/enteritis | R19.7; K52.1; K52.3; K52.8; K52.9; 558.9; 787.91 |
| Chemotherapy-induced fatigue | R53; 780.79 |
| Chemotherapy-induced nausea/vomiting | R11; 787.01; 536.2; 787.02; 787.03 |
| Chemotherapy-induced steatohepatitis | K75.81; K71.6; 571.8; 573.3 |
| Chemotherapy-induced neuropathy (and you may see peripheral neuropathy, sensor and motor neuropathy as well) | G62.X; G63.1; 357.7; 356.9; 355.9 |
| Chemotherapy-induced thrombocytopenia | D61.810; D69.59; 284.11; 287.49 |
| Chemotherapy-induced lung disease | J18.9, J84.89; 486; 516.8; 515 |
| Related Pain | G89.3; M25.50; 338.3; 338.29; 719.40 |
| Anemia due to chemotherapy | D64.81; D61.1; D63.0; 285.3; 284.89; 285.22 |
| Agranulocytosis secondary to cancer chemotherapy | D70. 1; D70.2; D70.8; D70.9; 288.50; 288.03; 288.00; 288.09 |
| Mouth sore secondary to chemotherapy | K12.30; K12. 31; K12.32; 528.00; 528.01; 528.02 |
| Dehydration/Hypovolemia | E86.X; 276.52; 276.51; 276.50 |
| Renal failure, drug induced | N14.1; N14.2; N14.4; 584.5 |
| Drug induced rash | L27.0, L27.1. L53.0, L27.8, L27.9, L56.0, L56.1; 693.0 |
| Infusion reaction | T80.X; V58.89; 999.9; 999.89; 999.88; 999.81; 999.80; 999.42; 999.33; 999.32; 999.31; 999.2; 909.3 |
| **IRAES** | |
| **Complication** | **ICD Codes** |
| Anemia | 285.3, 285.8, 285.9284.x, 283.x, D59.x, D61.x, D60.x, D64.2, D64.3, D64.8 |
| Thrombocytopenia | 287.3, 287.31, 287.8, 287.9, 287.31, 287.32, 287.4, 287.49, 287.5287.8, 287.9, D69.3, D69.41, D69.49, D69.59, D69.6 |
| Leukopenia | 288.00, 288.03, 288.09 288.4, 288.5x, 288.8, 288.9, D72.1, D72.81, D72.810, D72.818, D72.819, D70.9, D70.4, D70.2, D76.1, D76.3 |
| Hypothyroidism | 244.3, 244.8, 244.9, E03.2, E03.8, EO3.9 |
| Hyperthyroidism | 242.x, E05.x |
| Hypophysitis/PGA | 253.0, 253.1, 253.2, 253.4, 253.5, 253.6, 253.7, 253.8, 253.9, 255.0, 255.10, 255.11, 255.2, 255.3, 255.5, 255.41, 255.42, 255.5, 255.6, 255.8, 255.9, E22.x, E23.x, E27.x, E26.02, E26.09, E26.0, E26.1 E26.8, E26.89, E26.9, E24.x |
| Hyper/hypo- parathyroidism | 252.x, E21.x, E20.x |
| AKI | 584.x, 580.x, 581.x, 583.x N17.x, N00.x, N01.x, N04.x, N05.x N06.x, N14.1, N14.2 |
| Neuritis | 356.4, 356.8, 356.9, 357.4, 357.6, 357.7, 357.8, 357.9, 729.2, G60.3, G60.8, G60.9, G61.x, G62.0, G62.2, G62.9, G90.0, G90.0x, G90.2, G90.3, G90.4, M79.2 |
| Hepatitis | 573.3, 790.5, K71, R74.8, K75.4, K75.9, R94.5 |
| Colitis | 558.2, 558.3, 558.4, 558.9, 555, 556.8, 556.9, K52.1, 52.29, 52.3, 52.9, 52.89, 53.82 |
| Pancreatitis | 577.0, K85.x |
| Mucositis | 528.x, K12.30, K12.31 |
| Arrhythmia | 427.x I48.x, I47.x, I49.x, I46.x |
| Acute MI | 410.x, I21.x |
| Myocarditis | 422.x, 429.x, I40.x, I51.4, I51.8, I51. |
| Pericarditis | 420.x, 423.x, I30.x I31.4, I31.8-9 |
| Cardiomyopathy | 425.4, 425.9, I42.0, I42.7, I42.9 |
| Pneumonitis | 508.8, 508.9, 486.x, 516.3x, 516.9, J84.11x, J70.9, J70.8, J70.2, J70.3, J70.4, J18.9, J84.89, J84.9 |
| Type I diabetes | 250.0, 250.01, 250.03, 250.1, 250.11, 250.13, 250.2, 250.21, 250.23, 250.3, 250.31, 250.33, 250.4, 250.41, 250.43, 250.6, 250.61, 250.63, 250,7, 250.71, 250.73, 250.81, 250.83, 250.9, 250.91, 250.93, E09.x, E10.x, E13 |
| Meningitis | 047.9, 322.9, G03.9, A87.9 |
| Encephalitis, myelitis, encephalomyelitis | 323.8, 323.81, 323.82, 323.7, 323.71, 323.72, 323.9, G92, G04.81, G04.89, G04.90, G04.91 |
| Vitiligo | 709.00, 709.01, 709.09, L80, L81.8, L81.9, |
| **Psychological Affections** | |
| **Complication** | **ICD Codes** |
| Depression | F33, F32.89, F32.9, 311, 296.82, 296.33, 296.35, 296.30 |
| Anxiety | F41.9, F41.8, F41.1, 300.00, 300.4, 300.02 |
| Bipolar Disorder | F31, 296.80, 296.50, 296.89, 296.7 |
| **Other Complications** | |
| **Complication** | **ICD Codes** |
| Cognitive Decline/Dementia | 331.83, 294.20, 290.10, 290.11, 290.13, 290.21, 290.8, 799.52, 780.93, 294.9, 799.5, G31.84, R41.X, F03.90 |

**Supplemental Table II.** Cox proportional-hazard univariable and multivariable models accounting for association between race and treatment patterns or treatment adverse events for Breast Cancer University Hospitals (UH) population (2005-2022). Results are presented in hazard ratios (HR) for Blacks, lower 95% confidence intervals (L95), higher 95% confidence intervals (H95), and p-value.

|  | **Univariable** | | | | **Multivariable** | | | |
| --- | --- | --- | --- | --- | --- | --- | --- | --- |
|  | **HR** | **L95** | **H95** | **p value** | **HR** | **L95** | **H95** | **p value** |
| **Surgery (1)** | 1.07 | 1.02 | 1.13 | 0.003 | 0.92 | 0.87 | 0.97 | 0.004 |
| **Radiotherapy (2)** | 1.44 | 1.36 | 1.54 | <0.001 | 1.40 | 1.29 | 1.52 | <0.001 |
| **Hormone therapy (3)** | 0.96 | 0.9 | 1.01 | 0.18 | 0.83 | 0.79 | 0.89 | <0.001 |
| **Chemotherapy (4)** | 1.36 | 1.27 | 1.46 | <0.001 | 1.01 | 0.92 | 1.1 | 0.82 |
| **Chemotherapy complications (4)** | 1.15 | 0.91 | 1.46 | 0.23 | 1.13 | 0.85 | 1.49 | 0.38 |
| **Immunotherapy (5)** | 1.36 | 1.11 | 1.66 | 0.002 | 1.1 | 0.89 | 1.36 | 0.34 |
| **IRAES (5)** | 1.13 | 0.76 | 1.69 | 0.52 | 1.16 | 0.76 | 1.79 | 0.47 |
| **Psychological Affections (6)** | 0.94 | 0.86 | 1.03 | 0.21 | 0.71 | 0.63 | 0.80 | <0.001 |
| **Cognitive decline/dementia (7)** | 2.1 | 1.83 | 2.41 | <0.001 | 1.3 | 1.08 | 1.56 | 0.003 |
| **Sensitivity (>2015) (n=8,635)** | | | | | | | | |
|  | **Univariable** | | | | **Multivariable** | | | |
|  | **HR** | **L95** | **H95** | **p value** | **HR** | **L95** | **H95** | **p value** |
| **Surgery (1)** | 1.25 | 1.14 | 1.36 | <0.001 | 0.93 | 0.84 | 1.03 | 0.17 |
| **Radiotherapy (2)** | 1.23 | 1.11 | 1.36 | <0.001 | 1.11 | 1.01 | 1.25 | 0.04 |
| **Hormone therapy (3)** | 1.09 | 0.98 | 1.22 | 0.09 | 0.82 | 0.73 | 0.92 | 0.001 |
| **Chemotherapy (4)** | 1.71 | 1.50 | 1.95 | <0.001 | 1.02 | 0.87 | 1.19 | 0.74 |
| **Chemotherapy complications (4)** | 0.94 | 0.69 | 1.28 | 0.7 | 0.93 | 0.65 | 1.31 | 0.68 |
| **Immunotherapy (5)** | 1.61 | 1.22 | 2.13 | <0.001 | 0.89 | 0.64 | 1.22 | 0.48 |
| **IRAES (5)** | 0.77 | 0.45 | 1.31 | 0.33 | 0.8 | 0.43 | 1.48 | 0.48 |
| **Psychological Affections (6)** | 0.77 | 0.66 | 0.9 | 0.001 | 0.56 | 0.47 | 0.68 | <0.001 |
| **Cognitive decline/dementia (7)** | 1.95 | 1.52 | 2.51 | <0.001 | 1.5 | 1.13 | 1.97 | 0.003 |

| (1) Adjusted for age at diagnosis, histology, stage, Charlson, HER2 status, ER status, and PR status. |
| --- |
| (2)(4) Adjusted for age at diagnosis, smoking status, histology, stage, Charlson, HER2 status, ER status, and PR status. |
| (3) Adjusted for histology, stage, Charlson, ER status, and PR status. |
| (5) Adjusted for age at diagnosis, histology, stage, HER2 status, and ER status. |
| (6)(7) Adjusted for age at diagnosis, smoking status, histology, stage, Charlson, HER2 status ,ER status, PR status, chemotherapy, immunotherapy, surgery, radiotherapy, immunotherapy, and hormone therapy. |
